# Calcium ion currents mediating oocyte maturation events

**DOI:** 10.1186/1477-7827-4-26

**Published:** 2006-05-09

**Authors:** Elisabetta Tosti

**Affiliations:** 1 Cell Biology Laboratory, Stazione Zoologica, Naples, Italy

## Abstract

During maturation, the last phase of oogenesis, the oocyte undergoes several changes which prepare it to be ovulated and fertilized. Immature oocytes are arrested in the first meiotic process prophase, that is morphologically identified by a germinal vesicle. The removal of the first meiotic block marks the initiation of maturation. Although a large number of molecules are involved in complex sequences of events, there is evidence that a calcium increase plays a pivotal role in meiosis re-initiation. It is well established that, during this process, calcium is released from the intracellular stores, whereas less is known on the role of external calcium entering the cell through the plasma membrane ion channels. This review is focused on the functional role of calcium currents during oocyte maturation in all the species, from invertebrates to mammals. The emerging role of specific L-type calcium channels will be discussed.

## Review

### Oocyte maturation

Oogenesis is characterized by a unique process of cell division occurring only in gametes, called meiosis; whose goal is the production of haploid cells highly specialized for fertilization. In the majority of species the oocyte arrests in different stages of meiotic division, in particular, the block occurring in the first meiotic prophase (PI) marks the state of immature oocyte characterized by a prominent nucleus called the germinal vesicle (GV), which contains de-condensed transcriptionally active chromatin [[1] for a review]. As a general scheme, in response to a stimulus, meiosis is resumed and manifested by a germinal vesicle breakdown (GVBD), it then progresses to metaphase I (MI) or II (MII) where it undergoes a second arrest that is removed after successful fertilization.

Oocyte maturation is usually defined as the period of progression from the first to the second meiotic arrest and involves coordinated nuclear and cytoplasmic modifications [2]. These are highly complex processes and their interplay is regulated by a series of sequential molecular events. Nuclear maturation starts with the GVBD, ends at the meiosis exit, and is marked by the presence of the two polar bodies. Cytoplasmic maturation is a more obscure process and involves both morphological and functional alterations related to: i) changes in the expression profile of cell cycle control proteins responsible for driving the oocyte towards developmental competencies [3-5]; ii) relocation of organelles [6-8]; iii) transcriptional modifications of mRNA [9], modification of the plasma membrane permeability [10,11]; iv) the differentiation of the calcium signalling machinery [12].

**Figure 1:**
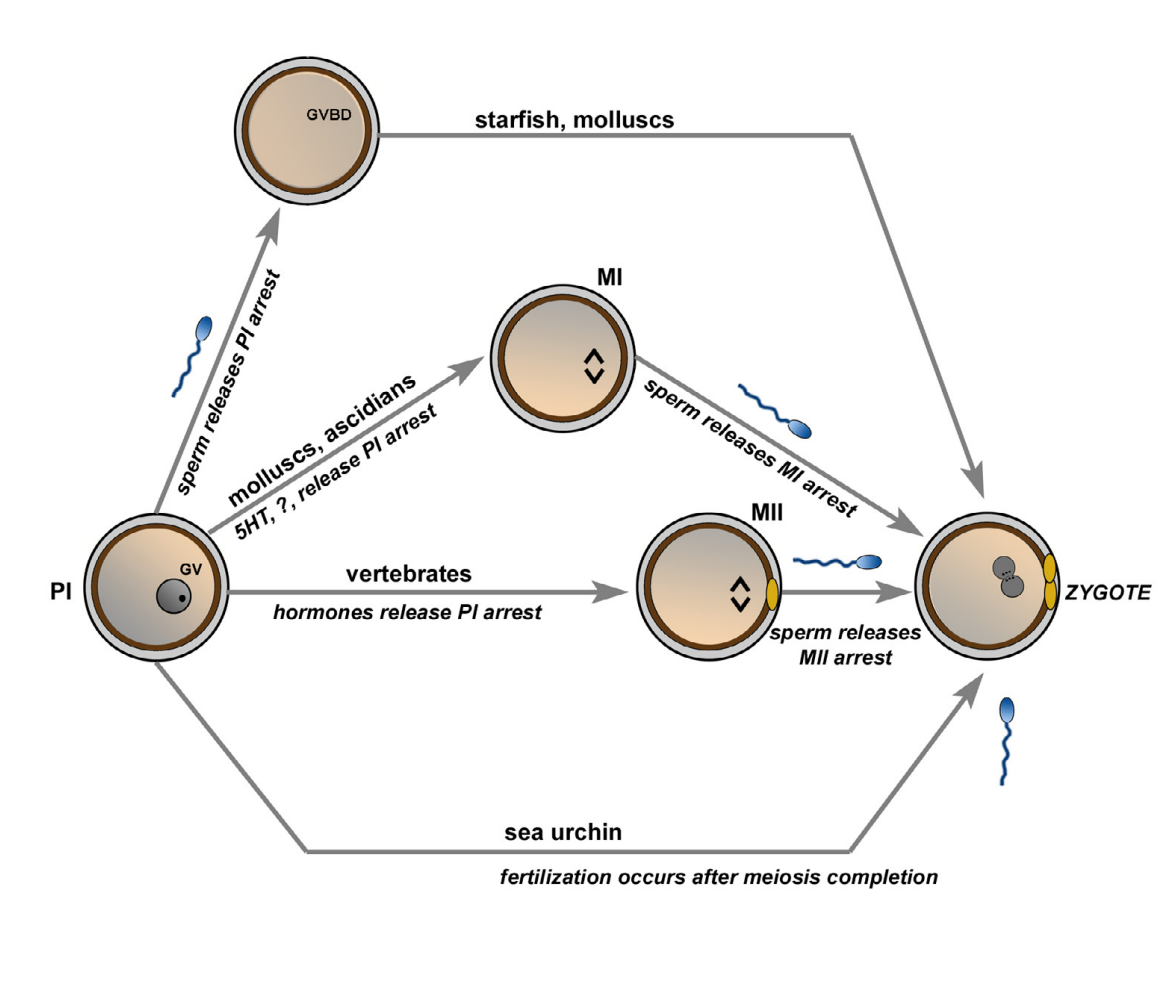
A schematic illustration of the resumption of meiosis in different animal models. The immature oocyte is arrested in prophase I (PI) marked by the germinal vesicle (GV). Depending on the species, oocytes may be fertilized in PI, undergoes a second meiotic block at the metaphase I (MI), metaphase II (MII) or may complete meiosis before fertilization. MII is marked by one polar body (yellow). Resumption from the second meiotic block occurs upon sperm penetration leading to germinal vesicle breakdown (GVBD), meiosis completion and zygote formation marked by the two inner pronuclei and two polar bodies.

Although the arrest at the PI stage seems to be strictly correlated with the oocyte growth, the meiotic stage correlated with fertilizable oocyte is species-specific. In some animals, oocytes are fertilized at the PI stage (anellida, plateyhelminthes, polychaeta, mollusca, arthropoda, echinoderms, and some mammals) or, on the contrary, there are some oocytes that are fertilized after meiosis completion (coelenterate, echinoderms). In worms, ascidians, molluscs, and some insects, a second arrest occurs at the MI stage and at the metaphase II (MII) stage in the Amphioxus and all the vertebrates [13,14] (Fig. 1).

The control of oocyte maturation involves a complex interplay between the oocyte and the extracellular membranes and the environment, with the participation of numerous metabolic pathways. The resumption of meiotic maturation relies on two different mechanisms: a positive stimulation and the removal of an inhibitory signal. The former involves the production of a ligand that acts on the oocyte at the GV stage inducing the GVBD. While this general mechanism is common to almost all the species studied, the nature of the ligand that allows the passage between the first and second meiotic block is different in each species.

Studies on this topic have established that 1-methyladenine (1-MA), serotonin, and steroids resume the first meiotic block in starfish [15], molluscs [11,16], fishes [17], and amphibians [18] respectively, while in mammals, it is the luteinizing hormone (LH) surge [19] that is known for initiating the transition from PI through MI to MII.

In the absence of positive stimulation, meiotic arrest appears to be maintained by a constraint of the environment surrounding the oocyte. In some species, oocytes undergo maturation as soon as they are isolated from their follicles or the external milieu, suggesting that these elements contain substances preventing meiosis resumption of PI arrested oocytes [20,21]. Assuming the existence of an interplay between the two mentioned mechanisms, meiosis resumption may occur through: i) the generation of a signal that in turn is transferred to the oocyte through the follicular environment; ii) the override of the environmental inhibition by removing the contact between oocyte and its follicular envelope and the closure of the connecting junctions [22].

Meiosis arrest and resumption are modulated by numerous messengers. Many studies have provided evidence of the involvement of cyclic nucleotides in the maintenance of meiotic arrest [23]. Elevated levels of cyclic adenosine mono-phosphate (cAMP), some analogues, cAMP-dependent protein kinase (PKA), and related substances such as GPR3, act by preventing spontaneous maturation and/or blocking GVBD in vitro [24-28]. However, contrasting data show that high levels of cAMP may only transiently block GVBD [29] or may even release the oocyte from meiotic arrest. [30,31].

Another important factor responsible for meiosis resumption is the M-phase promoting factor (MPF) showed for the first time in amphibian oocytes in the 70s, by Masui [32]. Nonetheless most of the work on MPF has been carried out with frog and starfish oocytes, accumulated evidence demonstrates that this complex function exists in other animal models, such as mammals and invertebrates [10,33-36] . Although oocytes from different species display different sensitivities to inhibitory and stimulatory ligands, there is a general consensus that calcium ions play a fundamental role in the resumption of meiotic maturation [4,37].

**Figure 2:**
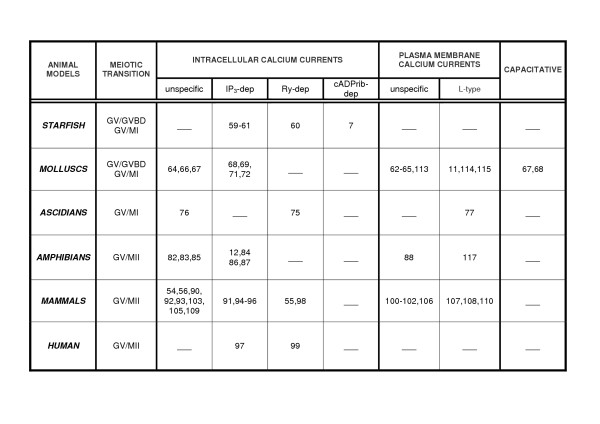
Review of the literature concerning the types of calcium currents involved in meiotic transition stages in oocytes of different animal models. Intracellular calcium currents may be mediated by IP_3_, Ryanodine (Ry), or cyclic ADP ribose. Plasma membrane calcium currents may be mediated by unspecific or L-type channels. Capacitative is the interplay mechanism that links plasma membrane and intracellular currents.

### Calcium and maturation

The role of ion currents in the oocyte physiology is described in many animal species [38-41]. In particular, calcium currents have been shown to be vital in regulating a broad range of physiological processes [42,43].

The calcium rise in the cell occurs by means of two principal mechanisms: the efflux from the stores via ligand-gated channels on organelle membranes, and the entry through ion channels in the plasma membrane. Most of the events underlined by the former mechanism are associated with two families of ion channels stored in the endoplasmic and/or sarcoplasmic reticulum in all cell types: the ryanodine receptor (Ryr) and the inositol 1,4,5-trisphosphate receptors (IP_3_r). The phosphoinositide pathway is of primary importance in mobilizing calcium into the cell, since elevation of IP_3 _levels elicits transient calcium currents from the intracellular stores [44,45]. On the other hand, calcium ionophore, is known to cause an increase in intracellular calcium concentration through Ryr. IP_3 _and Ry receptor/channels complexes share common features for what concerns both the amplification of calcium release by a positive feedback and the termination due to a negative feedback [46]. The responsiveness of the intracellular receptors/channels is regulated by a combination of factors, such as the calcium loading of the reticulum, and the sensitivity of the receptors to cytosolic calcium and to agonist concentration [46]. In excitable tissues, calcium entry is accomplished by the opening of voltage-operated calcium channels (VOCs) that mediate calcium influx in response to membrane depolarization [47]. At last, a connection between the two pathways is supported by the store-operated channels [48] through which a calcium influx is induced by the depletion of internal stores [49].

It is well established that calcium is involved in the physiology of the oocyte from oogenesis to maturation and fertilization [33,50-53]. Particularly, it has been described that the transition from one meiotic phase to the following is regulated by cell cycle control checkpoints which are in turn modulated by a transient increase of intracellular calcium in many animal species [4]. A general correlation between calcium and the GVBD has been demonstrated by a large number of studies. GVBD in mammalian oocytes is blocked by calcium chelators at least up to the first metaphase [33] whereas, in absence of intracellular calcium elevation spontaneous meiosis resumption in vitro does not occur [54]. Consistent data showed that injection of calcium in mouse oocytes induces parthenogenetic activation and subsequent normal development [55].

### Intracellular and plasma membrane calcium currents

Literature reveals that re-initiation of meiosis is mediated by both intracellular and plasma membrane calcium currents, sometimes in a synergic cooperation. In some species, intracellular currents stimulated by calcium ionophore induce oocyte maturation [56,57] whereas, particularly in starfish, it appears that IP_3_r amounts and sensitivity to IP_3 _increase during maturation. Although a direct correlation between GVBD and calcium internal currents has not been proven in this species [58], there is evidence that in the maturing oocyte the mechanism for calcium release is mediated also by Ry and cADP-ribose sensitive channels [7,59-61].

In molluscs in 1953, Allen [62] first reported a role of external calcium in the induction of GVBD in the *Spisula*. Later on, the external calcium requirement through voltage-gated channels was confirmed in this species and extended to the other molluscs that are also fertilized at the PI stage [63,64], or undergo the second arrest in MI [11,65]. Along with the extracellular calcium induction of GVBD in molluscs, it was soon recognized that there was an influence of the intracellular calcium elevation in almost all species studied independently from their peculiar meiotic arrest [64,66,67]. In particular, the interplay between external and internal calcium currents is evident in *Ruditapes*; here, a serotonin-induced surge of intracellular calcium was shown to trigger maturation even in the absence of external calcium [67]. As a general rule in molluscs, the initial plasma membrane calcium currents create a depolarization that, in turn, mobilize intracellular calcium currents from the stores [16,68]. However, a few exceptions must be mentioned, such as the *Hiatella flaccida*, where an intracellular calcium increase might be responsible for release from PI arrest, without a correlation with extracellular calcium [69]. Another example is the oyster [70], where calcium might not be involved in the early maturational stages. In surf clams and bivalves, experiments with IP_3_-induced GVBD suggest that release of internal calcium may be mediated by IP_3_-sensitive calcium currents [68,69,71,72].

Ascidians are ubiquitous marine invertebrates, whose oocytes maturate in the ovary. Immature oocytes are characterized by the GV; subsequently, to a still unknown stimulus, they undergo GVBD and resume meiosis up to the MI mature stage. Despite a large number of studies on ascidians, little information is available on the mechanisms that induce oocyte passage from the PI to MI block [73]. Very recently, Lambert [74] reviewed the signalling pathways underlying GVBD and he indicated that in some species the calcium ionophore induces GVBD [75]. In addition, it has been shown that intracellular calcium may either trigger or inhibit the GVBD onset [76]. Although these data show a general calcium role, a specific involvement of ion currents has been examined only recently in the ascidian *Ciona intestinalis*. Here, the first electrophysiological characterization of the plasma membrane at the GV stage oocytes – along with *in vitro *maturation experiments – strongly indicate a role of voltage-gated calcium currents in the prophase/metaphase transition [77].

Oocyte maturation mechanisms have been described in amphibians since the mid 80s [78]. Ion currents have been widely examined in immature oocytes of *Xenopus laevis *and *Rana esculenta *with growing evidence that chloride currents play a relevant role in the physiology of the oocyte [39]. Literature of the late 70s reports that transient calcium rises were associated to steroid-induced maturation events [79,80] proposing calcium function as the initial step in maturation induction. Although contrasting results indicated that calcium itself was not necessary to *Xenopus *oocyte maturation [81], recently Machaca [12] demonstrated a direct action of calcium release events during oocyte maturation in this species. Actually, evidence exists for an involvement of calcium currents in the activation of chloride [82-85], sodium, and hydrogen currents [86,87].

In amphibians, apart from a general change of membrane permeability during maturation [88], it seems that nobody has thus far correlated meiosis progression and/or GVBD to the intracellular or plasma membrane calcium current activity. However, when a role for ion calcium release in immature oocytes was shown, evidence demonstrated that this event occurs through IP_3_-sensitive stores currents [12,84,86,87].

In mammals, as a general scheme, oocyte maturation involves the resumption of meiosis in response to a surge of LH [23], the disruption of gap junctions after gonadotropin stimulation [89] and a decrease in cAMP levels [23]. Although a potential role of calcium currents in meiosis resumption is known, it remains to be elucidated if: i) calcium participates by itself as positive signal by coupling LH-induced GVBD or, ii) the other factors that traverse the gap junctions may influence the calcium levels within the oocyte. Literature shows that intracellular calcium oscillation is required for spontaneous maturation of mouse [90,91] and pig [92] oocytes, and that the increase in calcium concentrations at the time of GVBD confirms the relationship between intracellular calcium currents and oocyte maturation in different species [54,90,92,93]. The occurrence of spontaneous calcium oscillations in the mouse GV oocyte during meiotic maturation in vitro showed the involvement of an IP_3_-dependent mechanism [94], such as in hamsters [95], bovine [96], and humans [97].

Along with IP_3 _receptors and nonetheless many controversies, the occurrence of functional Ryr suggested an additional Ry-sensitive calcium-release mechanism in mouse [[55] and references therein], bovine [98], and human GV oocytes [99]. All together these data indicate that GV mammalian oocytes may account for both IP_3 _and Ry-mediated intracellular calcium currents in the meiotic transition from PI to MII stage.

Similar data have been reported for plasma membrane calcium currents; in fact the occurrence of both not-specific and calcium channels on the immature oocyte plasma membrane of mammals were demonstrated by Yoshida [100-102], whereas an externally derived calcium requirement at maturation was shown in the hamster [57] and other mammals [56,103-105].

In 1993, Murnane and De Felice [106] performed the first accurate electrophysiological characterization of immature murine oocytes demonstrating that plasma membrane calcium currents selectively increase in the growing oocyte and that this increase precedes nuclear maturation. These authors suggested that either intracellular or plasma membrane calcium currents may mediate the onset of oocyte maturation. In mice, confirming findings showed that GV and GVBD-arrested oocytes had some defects in calcium channel expression or translation, suggesting that an increase of calcium channel density may attain the oocyte meiotic competence [107].

The first electrophysiological characterization of GV stage bovine oocytes showed a plasma membrane calcium current activity during meiotic progression [108] and a prevalence of calcium stores at the GV stage [109]. Together these data indicated a possible association between LH-mediated calcium elevation and plasma membrane calcium currents. It was, in fact, suggested that in addition to store-released calcium, the plasma membrane currents might provide an alternative/additional mode of calcium entry in meiosis resumption. As it happens with bovine, recent preliminary experiments in sheep oocyte plasma membrane showed an involvement of calcium currents in the GV/MII transition [110]. Despite the general consensus, a few conflicting data show that calcium ion transport may underlie only a few phases of maturation [111] and even a calcium-independent GVBD in the mouse [112].

### L-type calcium currents

Numerous studies indicate that the intracellular calcium release is the universal mechanism that underlies the meiotic resumption at oocyte maturation [33,51]. On the contrary, the involvement of plasma membrane calcium currents has been described only in some species of molluscs [11,113-115], ascidians [77], amphibians [117], and mammals [106-108]. It is interesting that, in many cases, the specific channels involved in meiosis re-initiation are L-type calcium channels. These are voltage-gated channels that open in response to a depolarization of the plasma membrane and are expressed in different tissues in order to mediate signalling between cell membrane and intracellular processes, i.e. blood pressure regulation, smooth muscle contractility, insulin secretion, cardiac development, and learning and memory [[118], for a review]. In ascidians it was recently demonstrated that L-type calcium channels are involved in a series of biological processes [119]; however, first indication of a role of these channels in the reproductive processes was provided in mature oocytes [120,121], suggesting that cytosolic calcium release may be modulated by these plasma membrane calcium currents. Similarly, in some molluscs, progressive appearance of L-type calcium currents after stimulation by 5-HT correlated with the ability of MI-arrested oocytes seems to be responsive to fertilization [114,115]. Only in recent years has it been found that, in different species, oocyte maturation marked by the GVBD relies specifically on L-type calcium currents. In the mollusc this occurs in species with diverse maturational behaviour. In telolecitic oocytes of *Octopus vulgaris *maturation was strictly correlated with the decline in L-type calcium currents and the different developmental stages of cytoplasmic and nuclear maturation [11] and in the mussel oocytes a perfect correlation between inhibition of plasma membrane L-type calcium channels and inhibition of meiosis was shown [115]. In addition, in the *Mytilus *these channels appeared to be essential to sustain cytosolic calcium increase in order to extrude the first polar body.

A supporting finding also comes from the amphibians. Pleurodeles oocyte maturation is responsive to progesterone stimulation only during the breeding season versus a resting season. Interestingly, an electrophysiological study has strictly correlated the alternate expression of calcium channels in the two seasons, showing a higher current density and functional expression of the L-type during the maturational period. Furthermore, this study demonstrated a clear correlation of L-type calcium channel activity, cAMP levels, and the inability of the oocyte to mature [117]. In the ascidian *Ciona intestinalis *[77] the electrical characterization of the GV stage plasma membrane was recently carried out showing the higher occurrence of L-type calcium channels in the GV with respect to the mature stage. This pattern, together with the higher intracellular calcium release in the MI oocyte, has led to the hypothesis that L-type channels may play a double role in both regulating the GV/MI transition and participating in the loading of calcium stores necessary for subsequent fertilization. Similarly, the ability to reduce the GVBD in absence of external calcium further suggests that this response may require functional plasma membrane calcium channels [77].

Substantial differences occur in mammalian species. In the mouse it was first shown that the external calcium dependence implies the involvement of unspecific voltage-gated calcium channels in the onset of maturation in the different developmental stages such as oocytes-neonatal and GV stages [106]. However, a clear distribution pattern of the L-type calcium channels has only been subsequently provided showing that they undergo a density rearrangement only in the later stages of maturation until disappearing totally at the blastocyst stage [107].

Recently a significant distributional change of the L-type calcium channels activity from the GV to the MII stage was identified in bovine and ovine oocytes [108,110]. The results suggest that a possible common mechanism for the maturation starting in these two species is the calcium entry through specific channels potentiating the physiological oocyte-cumulus signalling responsible for meiotic awakening and progression.(Fig. 2)

## Conclusion

The evidence presented in this review supports the hypothesis that voltage-gated calcium ion currents are involved in the increase of cytosolic calcium levels occurring at oocyte maturation. Specific focus is centred on the occurrence and the pattern of L-type calcium currents during PI/metaphase transition in different animal species, implying that expression and translation of these types of calcium channels may be essential requirements for the oocyte maturation process and normal development. *In vitro *maturation of human oocytes is a challenge that could revolutionize the infertility treatment and IVF procedures. In this respect, future research will hopefully lead to determining the complex interplay between calcium current dynamics and other metabolic pathways participating in oocyte maturation aimed at successful oocyte fertilization and developmental competence.
